# Effects of different nitrogen application rates and picking batches on the nutritional components of *Lycium barbarum* L. fruits

**DOI:** 10.3389/fpls.2024.1355832

**Published:** 2024-04-24

**Authors:** Xiaojie Liang, Wei An, Yuekun Li, Xiaoya Qin, Jianhua Zhao, Shuchai Su

**Affiliations:** ^1^ Key Laboratory of Forest Silviculture and Conservation of the Ministry of Education, The College of Forestry, Beijing Forestry University, Beijing, China; ^2^ National Wolfberry Engineering Research Center, Wolfberry Science Research Institute, Ningxia Academy of Agriculture and Forestry Sciences, Yinchuan, China

**Keywords:** wolfberry fruits, nitrogen application rate, pick batch, nutrient composition, metabolomics

## Abstract

*Lycium barbarum* L., commonly known as wolfberry, is not only a traditional Chinese medicine but also a highly nutritious food. Its main nutrients include *L. barbarum* polysaccharide, flavonoid polyphenols, carotenoids, alkaloids, and other compounds, demonstrating its wide application value. This study investigated the effects of nitrogen application on the accumulation of the main nutrients and metabolites in wolfberry fruits under three different nitrogen application rates, namely, N1 (20% nitrogen (N) reduction, 540 kg·ha^–2^), N2 (medium N, 675 kg·ha^–2^), and N3 (20% nitrogen increase, 810 kg·ha^–2^,which is a local conventional nitrogen application amount.). Additionally, due to continuous branching, blossoming, and fruiting of wolfberry plants during the annual growth period, this research also explored the variation in nutritional composition among different harvesting batches. The contents of total sugar and polysaccharide in wolfberry fruit were determined by Fehling reagent method and phenol-sulfuric acid method, respectively;The content of betaine in fruit was determined by high-performance liquid chromatography,and the flavonoids and carotene in the wolfberry fruits were determined by spectrophotometry. Analysis of data over three consecutive years revealed that as nitrogen application increased, the total sugar content in wolfberry fruits initially decreased and then increased. The levels of *L. barbarum* polysaccharides, total flavonoids, and total carotenoids initially increased and then decreased, while the betaine content consistently increased. Different picking batches significantly impacted the nutrient content of wolfberry fruits. Generally, the first batch of summer wolfberry fruits had greater amounts of total sugar and flavonoids, whereas other nutrients peaked in the third batch. By employing a broadly targeted metabolomics approach, 926 different metabolites were identified. The top 20 differentially abundant metabolites were selected for heatmap generation, revealing that the contents of L-citrulline, 2-methylglutaric acid, and adipic acid increased proportionally to the nitrogen gradient. Conversely, the dibutyl phthalate and 2, 4-dihydroxyquinoline contents significantly decreased under high-nitrogen conditions. The remaining 15 differentially abundant metabolites, kaempferol-3-O-sophorosid-7-O-rhamnoside, trigonelline, and isorhamnosid-3-O-sophoroside, initially increased and then decreased with increasing nitrogen levels. Isofraxidin, a common differentially abundant metabolite across all treatments, is a coumarin that may serve as a potential biomarker for wolfberry fruit response to nitrogen. Differentially abundant metabolites were analyzed for GO pathway involvement, revealing significant enrichment in metabolic pathways and biosynthesis of secondary metabolites under different nitrogen treatments. In conclusion, a nitrogen application of 675 kg·ha^–2^, 20% less than the local farmers’ actual application, was most beneficial for the quality of four-year-old Ningqi 7 wolfberry fruits. Consumers who purchase wolfberry-dried fruit for health benefits should not consider only the first batch of summer wolfberry fruits. These results offer a broader perspective for enhancing the quality and efficiency of the wolfberry industry.

## 1 Introduction

Wolfberry (*Lycium barbarum* L.), a perennial deciduous shrub of the genus *L.*, Solanaceae, serves as a significant cash crop in Northwest China. It produces an infinite inflorescence, blooms, and fruits multiple times annually, and its fruit is highly valued for its rich nutritional and medicinal properties (Kulczyński and Gramza-Michałowska, 2016). Modern medical research has shown that wolfberry has various pharmacological effects, including effects on the lung, eye, kidney, and liver, as well as immunity enhancement, antiaging, antitumor, antifatigue, antioxidation, and synergistic anticancer effects (Khoo et al., 2017; Wang et al., 2018b; Yao et al., 2018). Wolfberry fruits are composed of key functional components such as total sugar, *L. barbarum* polysaccharide, betaine, carotenoids, flavonoids, polyphenols, and amino acids (Yajun et al., 2019). Sugars contribute significantly to the fruit’s sweetness and overall flavor, with moderately sweet fruits being preferred by consumers (Baldwin et al., 2000; Tieman et al., 2012). *L. barbarum* polysaccharide, a crucial active ingredient, enhances immune function (Zhou et al., 2018; Tian et al., 2019). Moreover, betaine, which function as a methyl donor, positively influences lipid metabolism and anti-fatty liver conditions (Christopher and Stephen, 2016). The carotenoids present in wolfberry, including β-carotene, lutein, lycopene, zeaxanthin, and zeaxanthin dipalmitate (Breithaupt et al., 2004), exhibit antioxidant properties and help prevent cancer, cardiovascular disease, and age-related macular degeneration (Islam et al., 2017). Flavonoids are known for their ability to relieve cough, expel phlegm, alleviate asthma, dilate coronary arteries, reduce blood cholesterol, strengthen cardiac contractions, and lower heart rate (Tanaka and Takahashi, 2013). The amino acid profile of wolfberry fruit includes 16 components, predominantly aspartic acid, glutamic acid, proline, serine, alanine, lysine, tyrosine, glycine, and 9 medicinal amino acids, such as leucine (Leu), aspartate (Asp), phenylalanine (Phe), glycine (Gly), lysine (Lys), methionine (Met), glutamic acid (Glu), arginine (Arg) and tyrosine (Tyr) (Zhang et al., 2004a). Additionally, wolfberry fruits contain γ-aminobutyric acid, hydroxyproline, citrulline, and other nonprotein amino acids with specific metabolic roles (Lu et al., 2021). Numerous organic acids and vitamins have also been identified in wolfberry fruits and roots, with studies indicating that wolfberry can provide twice the recommended daily dosage of vitamins A and C (Qian et al., 2017; Sun et al., 2019).

With the rapid advancement of the wolfberry industry, production challenges have become increasingly pronounced. Pursuing high yields and profits, farmers often disregard the actual nutrient requirements and resort to excessive chemical fertilizer use. Furthermore, unreasonable irrigation practices, which rely heavily on copious amounts of water and fertilizer, lead to suboptimal soil conditions, significant nutrient loss, reduced nitrogen fertilizer efficiency, declining economic returns, and increased risks of soil and groundwater pollution. This cultivation approach results in a detrimental cycle of high input and high yield, but low quality, impeding the green and sustainable development of the wolfberry industry. The chemical composition of medicinal plants is influenced by various environmental factors (Ng, 1999; Zhu et al., 2009). Although numerous reports exist on the pharmacological properties of wolfberry, the impact of fertilizer on the synthesis of bioactive substances in its fruit remains underexplored.

Nitrogen fertilizer plays a crucial role in crop growth, yield, and fruit quality (Chung et al., 2010). Proper nitrogen application enhances the soil nitrogen content, thereby promoting plant growth. However, excessive nitrogen use does not increase yield and can lead to environmental pollution (Ju et al., 2009). Several studies have examined the effects of nitrogen absorption and assimilation on plant growth and development (Kaplan et al., 2019). Research on wolfberry has indicated that nitrogen fertilizer significantly influences the growth of *L. barbarum* L. seedlings in spring, more so than phosphorus and potassium fertilizers (Shi et al., 2016). Furthermore, studies have identified the peak period for nitrogen uptake in wolfberry plants as early May to late June (Jianhong et al., 2008; Cai et al., 2013; Liu et al., 2016). Regarding the response of wolfberry fruit nutrients to nitrogen fertilizer, several researchers have reported that increasing nitrogen levels alter the main nutrients in wolfberry, with 53 metabolites (lipids, fatty acids, organic acids, and phenolic amides) exhibiting significant changes (Shi et al., 2019). Excessive nitrogen application can diminish the activities of betaine and betaine aldehyde dehydrogenase in wolfberry fruits, with the optimal nitrogen level for betaine accumulation being 450 kg/ha^2^ (Liu et al., 2020a). These findings lay a scientific foundation for understanding the response mechanism of wolfberry quality to nitrogen fertilizer. However, since wolfberry plants continue to branch, blossom, and bear fruit throughout the year, prior studies were largely based on a single fruit batch, and the changes in main nutrients across different batches under varying nitrogen conditions remain unreported.

To address this gap, this paper utilizes three years of field experiment data to examine the variations and differences in total sugar, *L. barbarum* polysaccharide, betaine, flavonoids, and carotenoids in wolfberry fruits under different nitrogen fertilizer treatments and in different batches. Concurrently, by employing an integrated LC-ESI-MS/MS detection system, the best quality wolfberry fruits from various batches were selected for analysis to explore the effects of different nitrogen treatments on wolfberry fruit metabolites. This study enhances the theoretical model and molecular mechanism underlying the response of wolfberry quality response to nitrogen fertilizer.

## 2 Materials and methods

### 2.1 Basic conditions of the experimental site and experimental design

The experiment was conducted at the base of Qiyuan Lvfeng Agriculture and Forestry Technology Co., Ltd., Sanhe Town, Haiyuan County, Zhongwei City, Ningxia (106°09’ 00” E, 36°25’ 48” N, altitude of 1428.5 m) from April 2020 until September 2022, with an average annual precipitation of 367 mm and an average annual temperature of 7.5°C. The terrain of the experiment was flat, with deep, high-quality, and uniformly fertile soils. The basic physical and chemical properties of the topsoil (0–20 cm) are shown in **Table 1**. The tested variety was four-year-old Ningqi No. 7 plants, the main variety of wolfberry. The selected test materials had great growth potential with no pests or diseases.

**Table 1:** Basic physical and chemical properties of the topsoil.

pH	total salt content (g·kg^–1^)	organic matter content (g·kg^–1^)	total nitrogen content (g·kg^–1^)	total phosphorus content (g·kg^–1^)	total potassium content (g·kg^–1^)	available nitrogen content (mg·kg^–1,^)	available phosphorus content (mg·kg^–1^)	available potassium content (mg·kg^–1^)
7.82	3.02	9.76	1.25	0.54	11.4	51.6	4.7,	118

The experiment followed a single-factor randomized block design, with each plot measuring 6 m×9 m and the plants spaced 1 m apart within rows that were 3 m apart. We conducted a one-way analysis of variance (ANOVA) with three treatments and five replications. The treatments included the following: ① N1 (low fertilizer): 20% nitrogen reduction, pure nitrogen of 540 kg·ha^-2^; ② N2 (medium fertilizer): medium nitrogen, pure nitrogen of 675 kg·ha^-2^; and ③ N3 (high fertilizer): increase nitrogen by 20%, pure nitrogen of N 810 kg·ha^-2^, which is a local conventional nitrogen application amount. The nitrogen (N), phosphorus (P), and potassium (K) fertilizers used were urea (N 46%), superphosphate (P_2_O_5_ 50%), and potassium sulfate (K_2_O 50%), respectively, and P and K fertilizers were applied at the same rate for each treatment, (450 kg·ha^–2^ and 300 kg·ha^–2^, respectively). According to the phenological period of wolfberry, these three fertilizers were applied in batches at 5 different fertilization times starting on approximately April 20 (the process of budding and unfolding), May 20 (the green fruit stage), June 15 (the fruit ripening process), late July to early August (the fruiting period in autumn), and late September (the resting period in autumn). Before May 20, the application rates of nitrogen fertilizer accounted for more than 50% of the annual application amount, while those of phosphorus and potassium fertilizers accounted for less than 40% of the annual application amount. After June, however, the application rates of nitrogen fertilizer accounted for less than 50% of the annual fertilizer amount, and those of P and K fertilizers accounted for more than 60% of the annual fertilizer amount. This approach resulted in the creation of nitrogen gradients among the various treatments. For field water management, we followed the traditional irrigation methods commonly used by local farmers. Irrigation was typically carried out approximately nine times throughout the growth period. The annual irrigation volume is approximately 7500 m^3^/ha^2^, of which the first irrigation occurs in mid to late April, the second irrigation occurs 8 to 10 days apart from the first, and the third irrigation occurs during the flowering and bearing period of perennial branches. During the summer harvest period, irrigation was carried out once every one to two fruit picking events, during which 2-3 irrigation events were generally carried out. After entering the autumn fruiting period, one irrigation event was carried out in early August combined with fertilization, one irrigation event was carried out in early September, and the last winter irrigation event was carried out in early November.

### 2.2 Determination of nutrients in wolfberry fruit

From 2020 to 2022, the first, second, and third batches of fresh wolfberry fruits will be picked at the summer fruit maturity stage every year, and the nutrient content will be determined after drying. All measurements were conducted in triplicate, and the average value was calculated. The contents of total sugar and *L. barbarum* polysaccharide were determined according to Appendix B of GB/T 18672-2014 (Wolfberry) (Zhang et al., 2014). The content of betaine in the fruit was determined by high-performance liquid chromatography (HPLC) with reference to Fang et al (Fang et al., 2011). Total flavonoid contents were determined by spectrophotometry, and rutin was used as the standard product for generating the standard curve according to the method of Zhang et al (Zhang et al., 2004b). The beta-carotene content was determined via in ultraviolet spectrophotometry using the method of Mi et al (Mi et al., 2018).

### 2.3 Extraction and quantitative analysis of metabolites from wolfberry fruit

#### 2.3.1 Extraction of metabolites

The freeze-dried samples were crushed using a mixer mill (MM 400, Retsch) with a zirconia beads for 1.5 min at 30 Hz. Then, 100 mg of powder was weighed, and pure methanol containing 0.1 mg·L^-1^ lidocaine was added for the extraction of fat-soluble metabolites (or 70% methanol was used for the extraction of water-soluble metabolites), vorticed once every 10 min three times, and then stored in a refrigerator at 4°C overnight. The next day, the sample was centrifuged (4°C, 10,000 rpm, 10 min), and the supernatant was collected. The water-soluble and fat-soluble metabolites were mixed 1:1, filtered through a microporous filter membrane (SCAA-104, 13 mm, 0.22 μm, ShanghaiAnpu Experimental Technology Co., Ltd., Shanghai, China, http://www.anpel.com.cn/15 November 2020) and stored in injection bottles for UPLC-MS analysis (Chen et al., 2014).

#### 2.3.2 HPLC conditions

The sample extracts were analyzed using an LC-ESI-MS/MS system (HPLC, Shim-pack UFLC SHIMADZU CBM30A system, Redwood City, USA www.shimadzu.com.cn/; MS, Applied Biosystems 6500 Q TRAP, www.appliedbiosytem.com.cn/). The analytical conditions were as follows. HPLC column, water ACQUITY UPLC HSSS T3 C18 (1.8 μm, 2.1 μm × 100 mm); solvent system, water (0.04% acetic acid), acetonitrile (0.04% acetic acid); gradient program, 95:5 v/v at 0 min, 5:95 v/v at 11.0 min, 95:5 v/v at 12.0 min, 95:5 v/v at 15.0 min; flow rate, 0.40 mL/min; temperature, 40°C; injection volume, 2 μL. The effluent was alternatively connected to an ESI-triple quadrupole-linear ion trap (Q TRAP)–MS instrument.

#### 2.3.3 ESI-Q TRAP-MS/MS

Mass spectrometry was performed according to the methods of Chen et al (Chen et al., 2013). Linear ion trap (LIT) and triple quadrupole (QQQ) scans were acquired on a triple quadrupole–linear ion trap mass spectrometer (Q TRAP), API 6500 Q TRAP LC/MS/MS System, equipped with an ESI Turbo Ion-Spray interface, which was operated in both positive and negative ion mode and controlled via Analyst 1.6 (AB Sciex, Concord, ON, Canada). The ESI source operation parameters were as follows: ion source, turbo spray; source temperature, 500°C; ion spray voltage (IS) 5500 V; and ion source gas I (GSI), gas II (GSII), and curtain gas (CUR),which were set at 55, 60, and 25.0 psi, respectively. The amount of collision gas (CAD) was high. Instrument tuning and mass calibration were performed with 10 and 100 µmol/L polypropylene glycol solutions in the QQQ and LIT modes, respectively. QQQ scans were performed as multiple reaction monitoring (MRM) experiments, with the collision gas (nitrogen) set to 5 psi. The declustering potential (DP) and the collision energy (CE) for individual MRM transitions were determined with further DP and CE optimization. A specific set of MRM transitions was monitored for each period according to the metabolites eluted within this period.

#### 2.3.4 Qualitative and quantitative analyses of the metabolites

Qualitative and quantitative analyses of metabolites followed the methods of Wang (Wang et al., 2018a) and Fraga (Fraga et al., 2010). Based on the self-built database MWDB (MetWare Biotechnology Co., Ltd. Wuhan, China) and the public database of metabolite information, qualitative analyses of the primary and secondary spectral data of mass spectrometry data were performed. The analyses removed the isotope signal, a repetitive signal containing K^+^ ions, Na^+^ ions, NH_4_^+^ ions, and a repetitive signal of fragment ions with larger molecular weights. The quantitative analysis of metabolites was performed using MRM analysis via QQQ mass spectrometry. After obtaining the metabolite mass spectrometry data of different samples, peak area integration was performed on the mass spectrum peaks of all substances, and the mass spectrum peaks of the same metabolite in different samples were integrated for correction.

### 2.4 Statistical analysis

The metabolite data were log2-transformed for statistical analysis to improve normality and were normalized. Metabolites from 9 samples were subjected to hierarchical clustering analysis (HCA), principal component analysis (PCA), and orthogonal partial least squares discriminant analysis (OPLS-DA) using R software to study metabolite accession-specific accumulation. The p and fold change values were set to 0.05 and 2.0, respectively. Venn diagrams were used to illustrate the number of differentially abundant metabolites. The Gene Ontology (GO) database with a p-value <0.01 was used to study differentially abundant metabolites associated with the different amounts of applied nitrogen. All the data were analyzed using Origin 9 (OriginLab Corporation, USA).

## 3 Results

### 3.1 Contents of the main nutrients in wolfberry fruits under different nitrogen application rates and batch picking conditions

#### 3.1.1 Total sugar content

**Figure 1:**
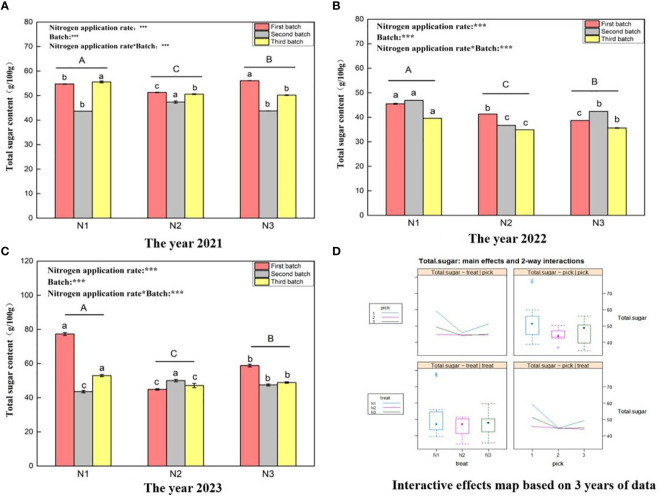
Variance analysis and interactive effect diagram of the total sugar content of wolfberry fruits. (**(A)** shows the total sugar data for fruits in 2021. The different capital letters in the figure indicate that the total sugar content of wolfberry fruits has significantly differed among the different nitrogen application rates after removing the picking batch factor (*P <* 0.05). Different lowercase letters in the figure indicate that the total sugar content of wolfberry fruits significantly differed among the different nitrogen application rates for the same picking batch (*P < 0.05*).In the upper left corner of the picture, the effects of the nitrogen application amount, picking batch, and nitrogen application amount in combination with the picking batch on the total sugar content in wolfberry fruits are shown. Two-factor analysis of variance was used, with *** indicating *P < 0.01* and ** indicating *P < 0.05.* The same applies below. **(B)** shows the total fruit sugar data for 2022. **(C)** shows the total fruit sugar data for 2023. **(D)** shows the interaction effect between the nitrogen application amount and harvest batch.).

**Figure 1**, shows that the total sugar content ranges of wolfberry fruits in 2021 (**Figure 1A**), 2022 (**Figure 1B**), and 2023 (**Figure 1C**) were 43.62–56.08 g/100 g, 34.9–46.9 g/100 g, and 43.57–77.33 g/100 g, respectively. This variation in the total sugar content of wolfberry fruits exhibits substantial year-to-year variability, likely linked to the annual climatic conditions. The results of one-way ANOVA showed that there were significant differences in the total sugar content of wolfberry fruits under different nitrogen application levels (*P < 0.05*), with N1 > N3 > N2 in descending order. This pattern remained consistent over the three years. Through a two-factor analysis of variance, it was determined that both the nitrogen application amount and the number of picking batches had notable effects on the total sugar content of wolfberry fruits. Additionally, an interaction effect was observed between the nitrogen application amount and the number of picking batches. According to the data from all three years, the first batch of fruits exhibited the highest total sugar content, while subsequent batches showed a decreasing trend. The total sugar content between the second and third batches was relatively similar (**Figure 1D**) upper left and lower left). Furthermore, under the N1 and N3 treatments, the total sugar content of the fruit was greater and displayed a trend of initially decreasing and then increasing with increasing nitrogen application (**Figure 1D** upper left and lower left).

#### 3.1.2 *L. barbarum* polysaccharide content

**Figure 2:**
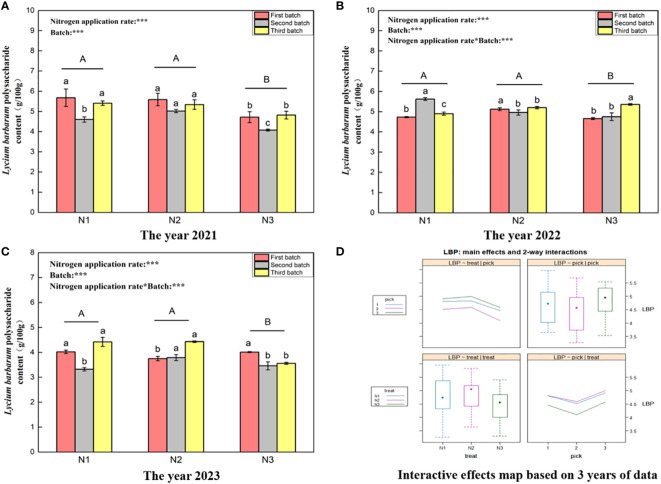
Variance analysis and interactive effect diagram of *L. barbarum* polysaccharides from wolfberry fruits. (**(A)** shows the *L. barbarum* polysaccharide data for fruits collected in 2021. The different capital letters in the figure indicate that the *L. barbarum* polysaccharide content of wolfberry fruits significantly differed among the different nitrogen application rates after removing the picking batch factor (*P <* 0.05).The different lowercase letters in the figure indicate that the *L. barbarum* polysaccharide content in wolfberry fruits significantly differed among the different nitrogen application rates in the same picking batch (*P < 0.05*).In the upper left corner of the picture, the effects of the nitrogen application amount, picking batch, and nitrogen application amount in combination with the picking batch on the *L. barbarum* polysaccharide content in wolfberry fruits are shown. Two-factor analysis of variance was used, with *** indicating *P < 0.01* and ** indicating *P < 0.05*. The same applies below. **(B)** shows the *L. barbarum* polysaccharide data for 2022. **(C)** shows the *L. barbarum* polysaccharide data for 2023. **(D)** shows the interaction effect between the nitrogen application amount and harvest batch.).

**Figure 2** shows that the *L. barbarum* polysaccharide content in wolfberry in 2021 (**Figure 2A**), 2022 (**Figure 2B**), and 2023 (**Figure 2C**) ranged from 4.08–5.68 g/100 g, 4.66–5.62 g/100 g, and 3.32–4.43 g/100 g, respectively. This wide variation in the *L. barbarum* polysaccharide content of wolfberry from year to year appears to be closely associated with the annual climatic conditions. One-way analysis of variance revealed no significant difference in the *L. barbarum* polysaccharide content between N1 and N2 under the different nitrogen application levels (*P > 0.05*). However, this content was significantly greater than that under the N3 treatment (*P < 0.05*), and this trend remained consistent over the three years. According to two-way analysis of variance, both different nitrogen application amounts and different picking batches had significant effects on the polysaccharide content of *L. barbarum*. Additionally, there was an interaction effect between nitrogen application amount and number of picking batches, except in 2021. According to the data from all three years, the *L. barbarum* polysaccharide content was highest in the third batch of fruits, exhibiting a trend of initial decrease and then increase with increasing number of batches. The content of *L. barbarum* polysaccharide was relatively similar between the first and third batches (**Figure 2D** upper right and lower right). Furthermore, the *L. barbarum* polysaccharide content in fruits was greater under the N1 and N2 treatments and initially increased and then decreased with increasing nitrogen application (**Figure 2D** upper left and lower left).

#### 3.1.3 Total flavonoid content

**Figure 3:**
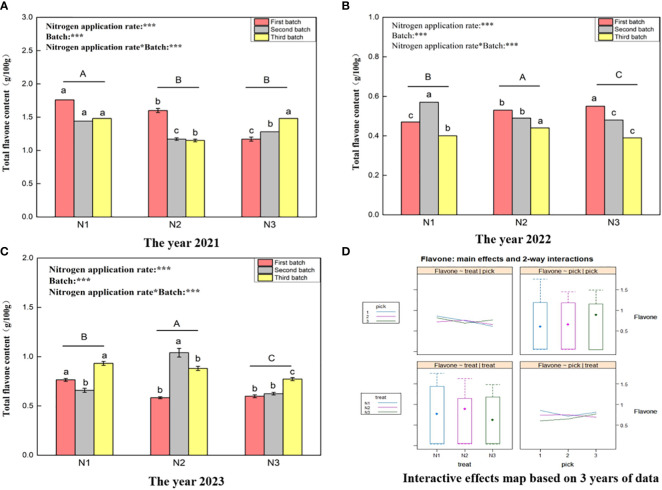
Variance analysis and interactive effect diagram of the total flavone content of the wolfberry fruits. (**(A)** shows the total flavone content data of fruits in 2021. Through one-way analysis of variance, different capital letters in the figure indicate that the total flavone content of wolfberry fruits significantly differed among the different nitrogen application rates after removing the picking batch factor (*P <* 0.05).The different lowercase letters in the figure indicate that the total flavone content of wolfberry fruits significantly differed among the different nitrogen application rates for the same picking batch (*P < 0.05*).In the upper left corner of the picture, the effects of the nitrogen application amount, picking batch, and nitrogen application amount in combination with the picking batch on the total flavone content in wolfberry fruits are shown. Two-factor analysis of variance was used, with *** indicating *P < 0.01* and ** indicating *P < 0.05*. The same applies below. **(B)** shows the total flavone content data for 2022. **(C)** shows the total flavone content data for 2023. **(D)** shows the interaction effect between the nitrogen application amount and harvest batch.).

**Figure 3** shows that the total flavone content ranges for wolfberry fruits in 2021 (**Figure 3A**), 2022 (**Figure 3B**), and 2023 (**Figure 3C**) were 1.15–1.76 g/100 g, 0.39–0.57 g/100 g, and 0.58–1.04 g/100 g, respectively. This substantial variation in the total flavone content of wolfberry fruits from year to year is evident. In 2021, under different nitrogen application rates, the total flavonoid content of wolfberry fruit was significantly greater under the N1 treatment than under the N2 and N3 treatments (*P < 0.05*). However, there was no significant difference between the N2 and N3 treatments (*P > 0.05*). In both 2022 and 2023, the total flavonoid content of wolfberry fruits initially increased and then decreased with increasing nitrogen application, and there were significant differences among all treatments (*P < 0.05*). Through two-way analysis of variance, it was determined that both different nitrogen application amounts and different picking batches had significant effects on the total flavonoid content of wolfberry fruits. Additionally, there was an interaction effect between nitrogen application amount and picking batch. According to the data from all three years, the total flavonoid content in wolfberry fruits was greater in the first batch of fruits, except for that in 2023. The total flavonoid content in fruits exhibited a decreasing trend with increasing batch size (**Figure 3D** upper right and lower right). Moreover, the content of total flavonoids in fruits was greater under the N1 and N2 treatments and followed a pattern of initially increasing and then decreasing with increasing nitrogen application (**Figure 3D** upper left and lower left).

#### 3.1.4 Betaine content

**Figure 4:**
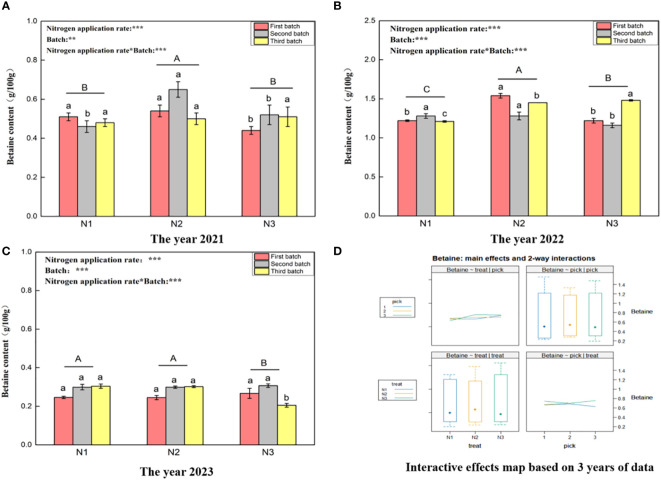
Variance analysis and interactive effect diagram of the betaine content of wolfberry fruits. (**(A)** shows the betaine content data for fruits collected in 2021. Through one-way analysis of variance, different capital letters in the figure indicate that the betaine content of wolfberry fruits significantly differed among the different nitrogen application rates after removing the picking batch factor (*P <* 0.05).Different lowercase letters in the figure indicate that the betaine content of wolfberry fruits significantly differed among the different nitrogen application rates for the same picking batch (*P < 0.05*).In the upper left corner of the picture, the effects of the nitrogen application amount, picking batch, and nitrogen application amount in combination with the picking batch on the betaine content in wolfberry fruits are shown. Two-factor analysis of variance was used, with *** indicating *P < 0.01* and ** indicating *P < 0.05*. The same applies below. **(B)** shows the betaine content data for 2022. **(C)** shows the betaine content data in 2023. **(D)** shows the interaction effect between the nitrogen application amount and harvest batch.).

**Figure 4** shows that the betaine content ranges for wolfberry in 2021 (**Figure 3A**), 2022 (**Figure 3B**), and 2023 (**Figure 3C**) were 0.44–0.65 g/100 g, 1.16–1.54 g/100 g, and 0.21–0.31 g/100 g, respectively. This significant variation in the betaine content of wolfberry from year to year is evident. In 2021, under different nitrogen application rates, the betaine content of wolfberry fruit was significantly greater under the N2 treatment than under the N1 and N3 treatments (*P < 0.05*). However, there was no significant difference between the N1 and N3 treatments (*P > 0.05*). In 2022, the betaine content of wolfberry plants under different nitrogen application rates significantly differed (*P < 0.05*), with the order from highest to lowest being N2 > N3 > N1. In 2023, the betaine content of wolfberry fruits under the N1 and N2 treatments did not significantly differ (*P > 0.05*), but it was significantly greater than that under the N3 treatment (*P < 0.05*). Through two-way analysis of variance, it was determined that both different nitrogen application amounts and different picking batches had significant effects on the betaine content of wolfberry fruits. Additionally, there was an interaction effect between nitrogen application amount and picking batch. Finally, based on data from three years, the betaine content in wolfberry fruits was highest in the third batch of fruits. The total betaine content in fruits increased initially and then decreased with increasing batch size (**Figure 4D** upper right and lower right). The betaine content in the fruit was greater under the N3 treatment and tended to increase with increasing nitrogen application rate (**Figure 4D** upper left and lower left).

#### 3.1.5 Total carotenoid content

**Figure 5:**
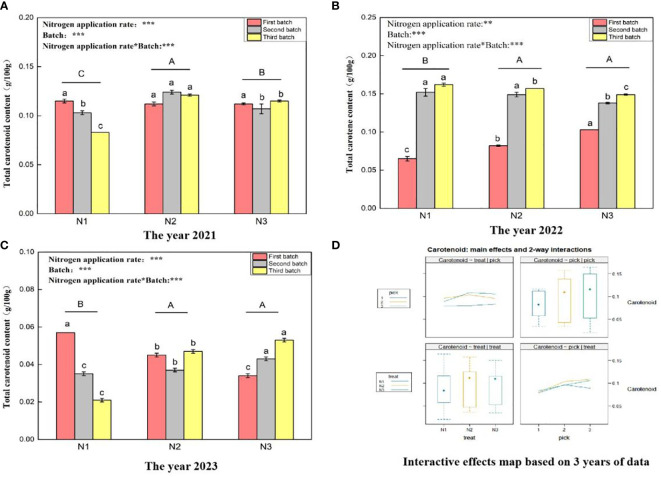
Variance analysis and interactive effect diagram of the total carotenoid content of wolfberry fruits. (**(A)** shows the total carotenoid content of fruits in 2021. Through one-way analysis of variance, different capital letters in the figure indicate that the total carotenoid content of wolfberry fruits significantly differed among the different nitrogen application rates for removing the picking batch factor (*P <* 0.05). Different lowercase letters in the figure indicate that the total carotenoid content of wolfberry fruits significantly differed among the different nitrogen application rates for the same picking batch (*P < 0.05*). In the upper left corner of the picture, the effects of the nitrogen application amount, picking batch, and nitrogen application amount in combination with the picking batch on the total carotenoid content in wolfberry fruits are shown. Two-factor analysis of variance was used, with *** indicating *P < 0.01* and ** indicating *P < 0.05*. The same applies below. **(B)** shows the total carotenoid content data for 2022. **(C)** shows the total carotenoid content data for 2023. **(D)** shows the interaction effect between the nitrogen application amount and harvest batch.).

**Figure 5** shows that the total carotenoid content ranges for wolfberry fruits in 2021 (**Figure 5A**), 2022 (**Figure 5B**), and 2023 (**Figure 5C**) were 0.083–0.124 g/100 g, 0.065–0.162 g/100 g, and 0.021–0.057 g/100 g, respectively. This considerable variation in the total carotenoid content of wolfberry fruits from year to year appears to be closely related to the annual climate conditions. In 2021, under the different nitrogen application rates, the total carotenoid content of wolfberry significantly differed among all the treatments (*P < 0.05*), with the order from highest to lowest being N2 > N3 > N1. In 2022 and 2023, there was no significant difference in the total carotenoid content between N2 and N3 under different nitrogen application levels (*P > 0.05*), but it was significantly greater than that in the N1 treatment group (*P < 0.05*). Through a two-way analysis of variance, it was determined that both different nitrogen application amounts and different picking batches had significant effects on the total carotenoid content of wolfberry fruits. Additionally, there was an interaction effect between nitrogen application amount and picking batch. Finally, considering data from three years, the total carotenoid content of wolfberry fruits was highest in the third batch of fruits. The total carotenoid content exhibited an increasing trend with increasing batch size (**Figure 5D** upper right and lower right). The total carotenoid content of the fruit was the highest under the N2 treatment, with a pattern of initial increase followed by a decrease with increasing nitrogen application rate (**Figure 5D** upper left and lower left).

### 3.2 Metabolomic analysis revealed the dynamic changes in metabolites in wolfberry fruits under different nitrogen application levels

**Figure 6:**
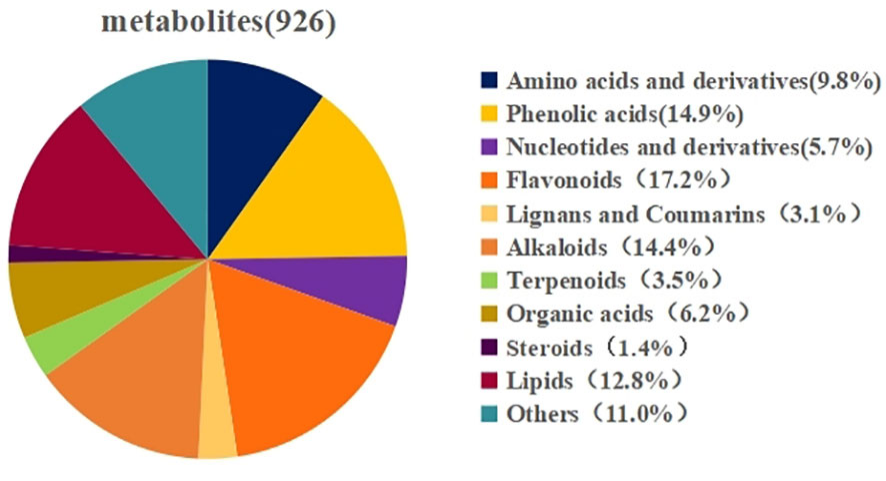
Metabolite classification diagram with annotated structure.

To gain a deeper understanding of the metabolite changes occurring in wolfberry fruits in response to different nitrogen application levels, we established a comprehensive metabonomics database using a broad targeted metabonomics approach facilitated by HPLC-MS technology. This database allowed us to identify primary and secondary metabolites within the samples. In total, 926 class I metabolites were detected, comprising 91 amino acids and their derivatives, 53 nucleotides and their derivatives, 57 organic acids, 119 lipids, 138 phenolic acids, 159 flavonoids, 29 lignans and coumarins, 13 steroids, 133 alkaloids, 32 terpenes, and 102 other compounds were detected (**Figure 6**).

**Figure 7:**
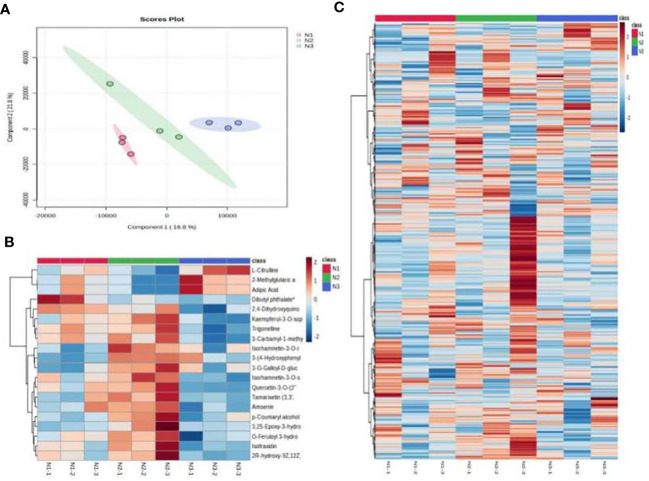
Metabolome analysis results for wolfberry fruits under different nitrogen application conditions. (**(A)** shows the PLS-DA analysis of different metabolites of wolfberry fruits under different nitrogen application conditions. **(B)** shows the clustering heatmap of the top 20 differentially abundant metabolites in wolfberry fruits under different nitrogen application conditions. **(C)** shows the total metabolite clustering heatmap of wolfberry fruit under different nitrogen application conditions).

The partial least square discriminant analysis of metabolites demonstrated that all samples fell within the 95% confidence interval without any outliers. Analysis of the point clouds from the three groups revealed significant differences in the metabolite profiles of wolfberry fruits subjected to low, medium, and high nitrogen application. Components 1 and Component 2 explained 16.8% and 21.8% of the variation, respectively (**Figure 7A**), underscoring substantial variations in metabolite accumulation among wolfberry fruits under different nitrogen application levels. To investigate the alterations in metabolites due to nitrogen application, heatmaps were generated to visualize metabolite contents in wolfberry fruits under various nitrogen conditions. The results highlighted the most pronounced changes in metabolites occurring under medium nitrogen application conditions (**Figure 7C**). We selected the top 20 differentially abundant metabolites to construct heatmaps, as illustrated in **Figure 7B**. Under low nitrogen application, the dominant metabolites in wolfberry fruits included dibutyl phthalate and 2,4-dihydroxyquinolines. In contrast, medium nitrogen application led to the dominance of metabolites such as kaempferol-3-O-sophoroside-7-O-rhamnoside, trigonelline, 3-carbamyl-1-methylpyridine oxide, isorhamnein-3-O-sophoroside, and 3-(4-hydroxyphenyl) propionic acid. High nitrogen application resulted in the prevalence of metabolites such as L-citrulline, 2-methylglutaric acid, and adipic acid in wolfberry fruits.

### 3.3 Multivariate analysis of wolfberry fruit metabolites under different nitrogen application rates

**Figure 8:**
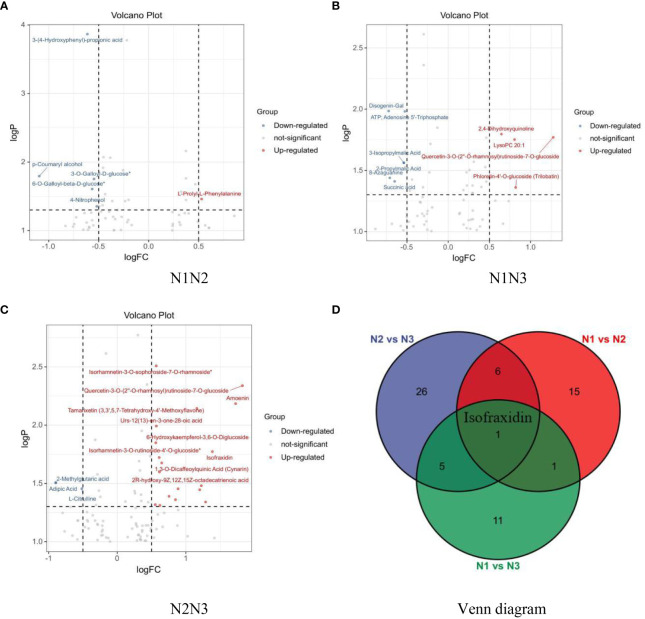
Volcano plot and Venn diagram of differentially abundant metabolites in wolfberry fruits under various nitrogen application conditions. (**A–C** show the volcanic plots of different metabolites in wolfberry fruits treated with N1N2, N1N3, and N2N3, respectively. **D** shows the Venn diagram of different metabolites of wolfberry fruits under different nitrogen application conditions.).

Univariate analysis quantified the magnitude of differences by calculating the fold change in metabolite levels. The screening criteria included *P* < *0.05* and a log2-fold change > *0.5* or < –*0.5*. As shown in **Figure 8A**, 63 different metabolites were identified in wolfberry fruit samples under low- and medium-nitrogen conditions, with 5 upregulated and 18 downregulated metabolites. **Figure 8B**, shows that 68 different metabolites were detected in wolfberry fruit samples subjected to low- and high-nitrogen application, with 7 upregulated and 11 downregulated metabolites. **Figure 8C** shows the 103 differentially abundant metabolites identified in wolfberry fruit samples under medium and high nitrogen application, including 28 upregulated and 10 downregulated metabolites. A Venn diagram (**Figure 8D**) was generated to analyze the differentially abundant metabolites in wolfberry fruits following pairwise comparisons across low, medium, and high nitrogen application levels. Notably, isofraxidin, a coumadin component, emerged as a common differentially abundant metabolite influenced by different nitrogen application levels.

### 3.4 Analysis of the metabolic pathways of wolfberry fruits under different nitrogen application rates

**Figure 9:**
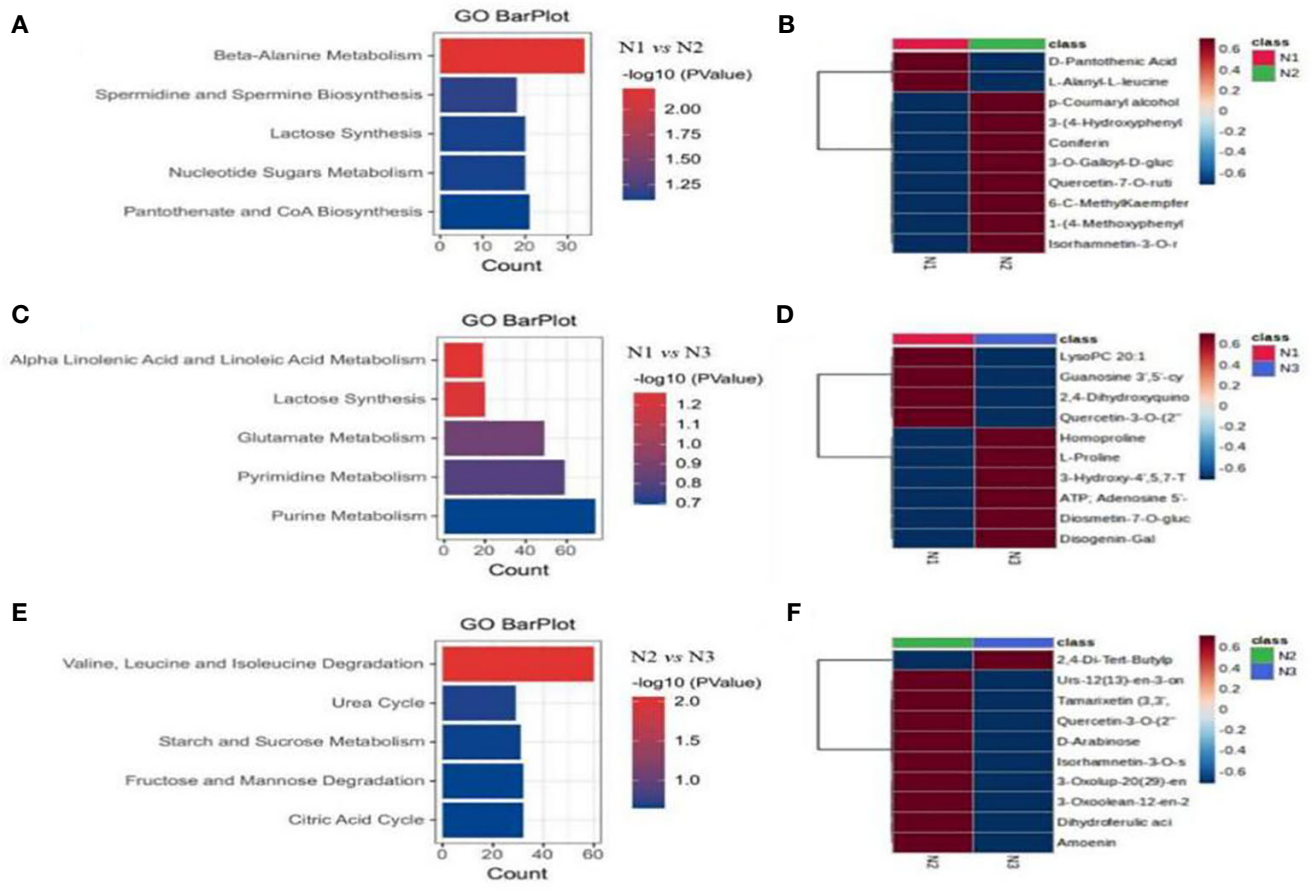
GO enrichment analysis of different metabolites of wolfberry fruits under different nitrogen application conditions. (**A–C** show the metabolic pathway enrichment of differentially abundant metabolites in wolfberry plants treated with N1N2, N1N3, and N2N3, respectively. **D–F** are the top10 differentially abundant metabolite clustering heatmaps of wolfberry fruits treated with N1N2, N1N3, and N2N3, respectively.).

We conducted GO pathway analysis on the differentially abundant metabolites to identify significantly enriched metabolic pathways. The most significantly enriched pathways in all three comparison groups were related to metabolic pathways and the biosynthesis of secondary metabolites (**Figures 9A–C**). Specifically, under low and medium nitrogen application levels, differentially abundant metabolites in wolfberry fruits were notably enriched in pathways such as β-alanine metabolism, spermidine and spermine biosynthesis, lactose synthesis, nucleotide sugar metabolism, and pantothenic acid and coenzyme A biosynthesis (**Figure 9A**). Moreover, under low and high nitrogen application levels, different metabolites were enriched in pathways such as α-linolenic acid and linoleic acid metabolism, lactose synthesis, glutamic acid metabolism, pyrimidine metabolism, and purine metabolism (**Figure 9B**). Finally, under medium and high nitrogen application conditions, differentially abundant metabolites in wolfberry fruits were significantly enriched in pathways involved in valine, leucine, and isoleucine degradation; the urea cycle; starch and sucrose metabolism; fructose and mannose degradation; and the citric acid cycle (**Figure 9C**). To further explore metabolites that exhibited significant changes in response to nitrogen application, we generated heatmaps for the 10 most significantly enriched metabolic pathways in each comparison group (**Figures 9D–F**). With the exception of D-pantothenic acid (vitamin B5) and L-alanyl-l-leucine, the expression levels of the other eight metabolites in N2 were significantly greater than those in N1 (**Figure 9D**). For N1 and N3, the expression of the first four differentially abundant metabolites (lysophosphatidylcholine 20:1, guanosine 3’,5’-cyclic monophosphate, 2,4-dihydroxyquinoline, quercetin-3-O-(2”-O-rhamnosyl) rutinoside 7-O-glucoside) was significantly greater in N1 than in N3 (**Figure 9E**). A comparison of the differentially abundant metabolites between N2 and N3 revealed that the expression levels of the other nine metabolites, except for 2,4-DBT phenol, were significantly greater in N2 than in N3 (**Figure 9F**).

## 4 Discussion

### 4.1 Effects of different nitrogen application rates on the nutrient content of wolfberry fruits

The primary goal of agricultural production has always been to enhance the yield and quality of cash crops. Adequate irrigation and nitrogen fertilizer application play pivotal roles in augmenting crop yield and quality. However, the relationships between crop yield, water, and nitrogen fertilizer follow a parabolic pattern. This means that when water and fertilizer levels exceed a certain threshold, crop yield and quality can be adversely affected (Sandhu et al., 2019; Liu et al., 2020b; Liu et al., 2021). In this study, the nutrient composition data of wolfberry fruits from 2020 to 2022 indicated that with increasing nitrogen application, the total sugar content in wolfberry fruits tended to initially decrease and then increase. The levels of *L. barbarum* polysaccharides, total flavonoids, and total carotenoids initially increased and then decreased, while the content of betaine continuously increased. These findings are generally consistent with prior research. For instance, Kang et al. reported that with increased nitrogen fertilizer application, the total sugar and betaine contents in wolfberry fruits tended to increase, while the *L. barbarum* polysaccharide and total carotenoid contents tended to decrease. However, the total flavonoid content contradicted the results of this study, as they observed an increasing trend with increasing nitrogen application (Kang et al., 2008). Cai et al.’s study revealed that the total sugar content of wolfberry fruits increased with nitrogen fertilizer application, but the *L. barbarum* polysaccharide content remained relatively stable with varying nitrogen levels (Cai et al., 2013). *L. barbarum* polysaccharide, a vital component of wolfberry fruits, comprises arabinose, glucose, galactose, mannose, xylose, and rhamnose. Most studies indicate that its content decreases with increased nitrogen application (Chung et al., 2010), suggesting that improving wolfberry fruit quality may involve sacrificing some biomass. Additionally, while the total sugar content in wolfberry fruit is an essential indicator, the trend in recent years has favored low-sugar foods due to improved living standards. Therefore, scientifically adjusting nitrogen fertilizer application to balance the total sugar and *L. barbarum* polysaccharide contents in wolfberry fruits may lead to the production of high-quality wolfberries with low total sugar and high polysaccharide contents. Regarding the change in flavonoid content with nitrogen application, Wei et al. reported that the total flavonoid content increased after nitrogen application, primarily due to the significant impact of nitrogen fertilizer on specific flavonoids such as 3’,4’,5’-tricetin O-rutinoside, chrysoeriol O-glucuronic acid-O-hexoside, tricin 7-O-hexosyl-O-hexoside, and tricin 5-O-hexoside (Shi et al., 2019). In contrast, in this study, the total flavonoid content increased initially and then decreased with increasing nitrogen application, suggesting that excessive nitrogen fertilizer application may have an inhibitory effect on flavonoid production. However, the precise mechanisms underlying this trend require further investigation. Betaine is one of the primary bioactive components in wolfberry fruits and is a quaternary ammonium compound (Chen and Murata, 2011). Its content correlates with the amount of applied nitrogen. Nonetheless, it has been observed that under conditions of insufficient nitrogen supply, wolfberry plants prioritize meeting their growth requirements over synthesizing the secondary metabolite betaine (Chung et al., 2010). In summary, adjusting the balance between yield and quality in wolfberry through nitrogen application is highly important for the high-quality development of the wolfberry industry, ensuring the quality of medicinal materials and maintaining stable clinical efficacy.

### 4.2 Effects of different picking batches on the nutrient content of wolfberry fruits

Wolfberry fruits are categorized into summer and autumn fruits based on their picking period. Summer fruits typically ripen from mid-June to the end of August, with the highest yield in the third batch, which best represents the quality of summer fruits. Autumn fruit ripening usually occurs from mid-September to the end of October (Zhang et al., 2007). Due to the high cost of manually picking autumn fruits, most farmers do not harvest them. In this experiment, the first three batches of summer fruits were collected under different nitrogen application conditions for three consecutive years, after which the primary nutrients in the dried wolfberry fruits were determined. The results revealed that the first batch of fruits had the highest total sugar and total flavonoid contents, while other nutrients reached their maximum levels in the third batch of summer fruits. Few studies have explored the impact of different picking batches on the nutrient content of wolfberry fruits. Previous research has shown that the polysaccharide content of the first batch of wolfberry fruits, characterized by higher *L. barbarum* polysaccharide content, exceeded that of summer fruits by approximately 25.3% (Ye and Zhao, 2004). Zhang et al. reported that different picking periods significantly affected the contents of *L. barbarum* polysaccharides, total sugars, amino acids, and betaine, with the first batch exhibiting higher levels than the other batches (Zhang et al., 2007). In light of these findings, it can be inferred that the first batch of wolfberry fruits exhibits certain advantages in terms of nutrient content after three months of winter and three months of spring. However, multiple factors contribute to the quality of wolfberry fruit and the nutritional value of other batches of summer fruits should not be underestimated through artificial cultivation measures. Furthermore, previous research on the first batch of wolfberries revealed that their appearance and palatability did not match those of other summer fruit batches (Ye and Zhao, 2004), suggesting that consumers should not blindly favor the first batch when purchasing functional health products to avoid unsold wolfberry fruits in other batches.

### 4.3 Metabolic characteristics of wolfberry fruits under different nitrogen fertilizer treatments

Metabolites serve as the foundation of biological phenotypes and offer a more intuitive and effective means to comprehend biological processes and mechanisms (Shi et al., 2019).In this study, a comprehensive total of 926 different metabolites were identified under three distinct nitrogen application treatments via a broadly targeted metabolomics approach. Among them, 309 metabolites were detected in N1, 434 in N2, and 183 in N3. In comparison to those in the N1 and N3 treatments, most metabolites in the N2-treated fruits were upregulation. Notably, the dominant metabolites in the N2 treatment group included kaempferol-3-O-sophoroside-7-O-rhamnoside, trigonelline, 3-carbamoyl-1-methylpyridine oxide, isorhamnein-3-O-sophoroside, and 3-(4-hydroxyphenyl) propionic acid, which are primarily flavonoids or alkaloids. Flavonoids are a valuable group of secondary metabolites in plants and are known for their various beneficial properties, including anticancer, anti-inflammatory, antioxidant, and bone-strengthening effects (Dong et al., 2009; Zhao et al., 2019). Wolfberry contains thirteen different flavonoid compounds, with rutin, quercetin, and kaempferol being the main flavonoid metabolites. In recent research, an additional seven flavonoid compounds have been identified in wolfberry fruits, expanding the repertoire of flavonoids present in wolfberry (Yang et al., 2022). The differentially abundant metabolites of wolfberry fruits subjected to N1, N2, and N3 treatments were analyzed using a Venn diagram, revealing that isofraxidin was a common differentially abundant metabolite influenced by different nitrogen application levels. Isofraxidin is a coumarin-like substance, and coumarins have gained significant attention in recent years for their diverse physiological activities, including anticancer, antioxidation, anti-inflammation, anti-HIV, anticoagulation, antibacterial, analgesic, and immune-regulating properties (Wu et al., 2009). As isofraxidin was the sole common differentially abundant metabolite across all treatments in this study, coumarins may serve as potential biomarkers for the response of wolfberry fruits to nitrogen. The differences in GO enrichment classification under various nitrogen fertilizer treatments primarily pertain to metabolic pathways and the biosynthesis of secondary metabolites. Specifically, the N1 and N2 treatments were predominantly associated with β-alanine metabolism, spermidine and spermine biosynthesis, lactose synthesis, nucleotide sugar metabolism, and pantothenic acid and coenzyme A biosynthesis. Moreover, under N1 and N3 conditions, the focus shifted to α-linolenic acid and linoleic acid metabolism, lactose synthesis, glutamic acid metabolism, pyrimidine metabolism, and purine metabolism. Finally, the N2 and N3 treatments were primarily associated with the degradation of valine, leucine, and isoleucine; the urea cycle; starch and sucrose metabolism; fructose and mannose degradation; and the citric acid cycle.

## 5 Conclusions

In summary, the application of 675 kg·ha^–2^ of nitrogen, which is 20% lower than the local farmers’ actual nitrogen application rate, was most beneficial for obtaining high-quality wolfberry fruit from four-year-old Ningqi 7 plants, a major wolfberry variety. Analysis of three years of field experiment data revealed that both the nitrogen application amount and picking batch significantly affected the main nutrient content in wolfberry fruits. A total of 926 metabolites were identified in wolfberry fruits subjected to the N1, N2, and N3 treatments, with 65 metabolites significantly influenced by nitrogen application. Isofraxidin, a coumarin-like substance, emerged as a common differentially metabolite across all treatments, suggesting its potential as a biomarker for wolfberry fruit response to nitrogen environments.

## Data availability statement

The original contributions presented in the study are included in the article/supplementary material. Further inquiries can be directed to the corresponding author.

## Author contributions

XL: Data curation, Investigation, Writing – original draft. WA: Writing – review & editing. YL: Investigation, Writing – review & editing. XQ: Investigation, Writing – review & editing. JZ: Data curation, Writing – original draft. SS: Writing – review & editing.
